# Acute Physiological Stress Down-Regulates mRNA Expressions of Growth-Related Genes in Coho Salmon

**DOI:** 10.1371/journal.pone.0071421

**Published:** 2013-08-19

**Authors:** Toshiki Nakano, Luis O. B. Afonso, Brian R. Beckman, George K. Iwama, Robert H. Devlin

**Affiliations:** 1 Marine Biochemistry Laboratory, Graduate School of Agricultural Science, Tohoku University, Sendai, Japan; 2 Institute for Marine Biosciences (IMB/NRC), National Research Council Canada, Halifax, Nova Scotia, Canada; 3 Northwest Fisheries Science Center, National Oceanic and Atmospheric Administration (NOAA), Seattle, Washington, United States of America; 4 The University of Northern British Columbia, Prince George, British Columbia, Canada; 5 West Vancouver Laboratory, Center for Aquaculture and Environment Research (CAER- DFO/UBC), Fisheries and Oceans Canada, West Vancouver, British Columbia, Canada; Centre of Marine Sciences & University of Algarve, Portugal

## Abstract

Growth and development in fish are regulated to a major extent by growth-related factors, such as liver-derived insulin-like growth factor (IGF) -1 in response to pituitary-secreted growth hormone (GH) binding to the GH receptor (GHR). Here, we report on the changes in the expressions of *gh*, *ghr*, and *igf1* genes and the circulating levels of GH and IGF-1 proteins in juvenile coho salmon (*Oncorhynchus kisutch*) in response to handling as an acute physiological stressor. Plasma GH levels were not significantly different between stressed fish and prestressed control. Plasma IGF-1 concentrations in stressed fish 1.5 h post-stress were the same as in control fish, but levels in stressed fish decreased significantly 16 h post-stress. Real-time quantitative PCR (qPCR) analysis showed that *ghr* mRNA levels in pituitary, liver, and muscle decreased gradually in response to the stressor. After exposure to stress, hepatic *igf1* expression transiently increased, whereas levels decreased 16 h post-stress. On the other hand, the pituitary *gh* mRNA level did not change in response to the stressor. These observations indicate that expression of *gh*, *ghr*, and *igf1* responded differently to stress. Our results show that acute physiological stress can mainly down-regulate the expressions of growth-related genes in coho salmon *in vivo*. This study also suggests that a relationship between the neuroendocrine stress response and growth-related factors exists in fish.

## Introduction

In fish, growth is regulated to a major extent by liver-derived insulin-like growth factor (IGF) -1 in response to pituitary-secreted growth hormone (GH) binding to GH receptors (GHR) of liver, and such GH-IGF-1 axis plays an important and critical role in the regulation of both growth and development. Secretion of GH is known to be under hypothalamic regulation by means of many modulators such as somatostatin, GH-releasing hormone, dopamine, and ghrelin [1]–[7]. The actions of IGFs are controlled by GH and IGF binding protein (IGFBP) and a specific receptor on the surface of target cells. The liver is the primary organ which produces IGF-1, though locally produced IGF-1 in organs other than the liver has been detected and may act in an autocrinal or paracrinal manner [2], [4], [7]–[9].

Growth in fish is genetically regulated and is also influenced by cellular, endocrinological, and environmental factors. The response of endocrinal tissue is affected by the integration of external stimuli with internal signals based on physiological status [2], [6]–[16]. Therefore, growth can be enhanced by elevated temperature, improved nutrition, husbandry conditions, and changes in the endocrine system of the animal [10], [12], [17]. Furthermore, growth can be significantly stimulated by treatment with exogenous GH in many fish [17]. Recently, *gh* transgenes have been transferred to fish with strong stimulation of growth [18], [19]. In *gh* transgenic salmon, the levels of GH and IGF-1 in plasma and mRNA expressions of *igf1* and *ghr* were also observed to increase [20], [21].

Cultured fish are exposed to many stressors from their environment and the chances of succumbing to infectious diseases may be increase a result [12], [13], [15], [16], [22]–[24]. In response to a stressor, a series of biochemical and physiological changes occur at the cellular and organismal levels. These stress responses can affect organismal conditions such as health, disease resistance, growth, and reproduction in fish [11], [12], [15], [16], [25], [26]. While the levels of growth-related factors in fish have often been discussed with nutritional and osmotic changes [2], [7]–[9], [11], [12], [14], [27], [28], little attention has been paid to the effect of an acute and non-severe stressor, such as physical disturbance, on the expression levels of gene and gene-products related to growth in fish [6], [7], [15], [28]. It is important to elucidate the feature and mechanism of the influence of physiological stress on fitness at both molecular and organismal levels for the improvement of production and health of fish under cultured conditions.

Coho salmon (*Oncorhynchus kisutch*) is known to be an important fish species for economics and production of food. Especially in Japan, coho salmon farming is one of the basic industries in northeastern (Tohoku) Pacific coastal area, Sanriku coast, where the great earthquake and tsunami occurred in 2011. Therefore, it has been desired to improve and increase a productivity of coho salmon farming for reconstruction of coastal fisheries in disaster-stricken area.

In this study, we examined changes in mRNA expression levels of *gh*, *ghr*, and *igf1* genes in response to an acute physiological stress derived from physical disturbance, handling, in juvenile coho salmon. We chose handling as a physiological stressor for fish in this study, because the handling stress is one of most common stress in aquaculture [11], [13], [25]. The circulating levels of GH and IGF-1 hormones in plasma were also determined. We discuss the relationships among the expressions of growth-related factors, stress response, and corticosteroids in fish in the context of our findings.

## Materials and Methods

### Ethics Statement

All the protocols of fish treatment were approved by the Fisheries and Oceans Canada Pacific Region Animal Care Committee (Nanaimo, BC, Canada).

### Fish, Rearing Conditions, Stress Performance, and Sampling

Coho salmon, Chehalis River (BC, Canada) broodstock, were raised in 3000L tanks supplied with well water in Center for Aquaculture and Environment Research (CAER)’s Aquarium Facility (West Vancouver, BC, Canada). The fish were fed by hand to apparent satiation twice a day with commercial feed (Skretting Canada, Canada). Healthy, mixed sex, juvenile fish were divided into 3 groups (n = 8). The fish in the first group were undisturbed (prestressed) fish used as a control, maintained under quiet and suitable conditions, and sampled at 9∶00. The fish in the second group were subjected to the stressor at 8∶30 and sampled at 1.5 h post-stress (at 10∶00). The fish in third group were also subjected to the stressor at 17∶30 and sampled at 16 h post-stress (at 9∶30). Accordingly, all tissues and blood for analysis were sampled at almost the same time in the morning, so that the effects of several factors, such as diurnal rhythm and photoperiod, on the expressions of growth-related factors should be minimized.

Four fish were placed into each of 6 separate 180 L fiber glass tanks supplied with running 10–11°C well water and acclimated to laboratory conditions under natural photoperiod for 5 days prior to experiments. Food was withheld for 24 h prior to the experiment. Nutritional status, such as starving, has been reported to influence the GH-IGF-I axis in fish, but 2 to 4 weeks of starving was required to obtain a significant change in the expression of growth-related factors in fish [7], [8], [27]–[30]. Accordingly, the short-term starving performed in the present study should not affect the expressions of growth-related factors in coho salmon.

Fish, with a body weight of 23.9±3.5 g (mean ± SD), were subjected to physical disturbance both from a 2 min of chasing by a hand-held dip net followed by a 0.5 min emersion from the water held within in the dip net. All stressed fish were returned to control conditions after this treatment. To minimize the effect of sampling protocol, five fish were sampled from each experimental tank at each sample time, thereby avoiding repeated sampling from the same tank. Total number of fish sampled at each time was 8 per group. Fish were anaesthetized with 100 mg/L tricane methane sulphonate (MS-222) buffered with 100 mg/L sodium bicarbonate and rapidly team-sampled for blood and tissues. Blood was collected from the caudal vein with using a heparinized syringe and plasma was separated by centrifugation at 1,000×*g* for 10 min, stored frozen at −80°C. All tissues were immediately immersed in RNA later (Ambion-Life Technologies, Austin, TX) and then stored at −80°C until later analyses.

### Measurements of Cortisol and Glucose Levels in Plasma

Plasma cortisol levels were measured using a commercially available enzyme-linked immunosorbent assay kit from Neogen Corp. (Lexington, KY) [31].

Plasma glucose was measured using an enzymatic-glucose oxidase assay method available in kit (Sigma-Aldrich, St. Louis, MO) [32].

### Measurements of GH and IGF-1 Levels in Plasma

Circulating levels of both GH and IGF-1 were analyzed using a chloramine-T iodination (^125^I)-based radioimmunoassay (RIA) established by Swanson [33] and Moriyama et al. [34], respectively.

In cases where plasma GH levels were below the detectable level, the assay’s lower detection limit of 0.8 ng/ml was used as values.

Plasma IGF-1 was determined using components (IGF-1 and anti-IGF-1 antibody) from GroPep (Adelaide, Australia). Plasma samples for IGF-1 were extracted with acid ethanol to remove binding protein effects.

### RNA Extraction and cDNA Synthesis

Tissues were placed in TRIzol reagent (Invitrogen-Life Technologies, Carlsbad, CA) and immediately homogenized using a polypropylene pestle. The resulting RNA pellet was dissolved in RNase-free water (UltraPure, Gibco-Life Technologies, Grand Island, NY) and quantified by spectrophotometry (Spectronic 1001 PLUS, Milton Roy, Ivyland, PA) before being diluted to 500 ng/µl to be used in reverse transcription reactions. RNA samples were stored at -80°C. Complementary DNA (cDNA) was synthesized using the Multiscribe Reverse Transcriptase Kit (Applied Biosystems-Life Technologies, Foster City, CA) with random hexamer primer or with a gene specific primer for salmon GH (*gh*-reverse primer, Table 1), and 250 ng RNA [20].

**Table 1:** Primers and probes used for the quantitative real-time PCR (qPCR) analysis in this study.

Gene	Forward Primer (5′–3)	Reverse Primer (5′–3′)	TaqMan Probe (5′–3′)
*gh*	CAAGATATTCCTGCTGGACTT	GGGTACTCCCAGGATTCAATCA	CAGTCCTGAAGCTGC
*ghr*	CACTGTGGAAGACATCGTGGAA	CAAAGTGGCTCCCGGTTAGA	AACTGGACCCTGCTGAA
*igf1*	GGCATTTATGTGATGTCTTCAAGAGT	CCTGTTGCCGCCGAAGT	TCTCACTGCTGCTGTGC
*ß-actin*	ACGGCCGAGAGGGAAATC	CAAAGTCCAGCGCCACGTA	CACAGCTTCTCCTTGATGT

### Determination of *gh*, *ghr*, *igf*, and *β-actin* mRNA Levels

The levels of *gh*, *ghr*, and *igf1* mRNA expressions in the tissues were determined by real-time quantitative PCR (qPCR) with an equipment of ABI Prism 7000 Sequence Detection System (Applied Biosystems-Life Technologies, Foster City, CA) using *β-actin* as an internal standard according to Raven et al. [20]. Primers and TaqMan probes for *β-actin*, *gh*, *ghr*, and *igf1* genes were designed from alignments of teleost cDNA sequences from GenBank to allow amplification using conserved regions (Table 1) [20], [35]. Values for *gh*, *ghr*, and *igf1* were normalized with those of *β-actin*. Levels of *β-actin* did not change with respect to treatment. Accordingly, each sample amplification value for each gene was expressed as a relative gene expression ratio (relative mRNA value).

### Statistical Analysis

All samples were run in duplicate and results are reported as mean ± SEM. All data were subjected to one-way analysis of variance (ANOVA). Means were compared with Fisher’s least-square difference (LSD) multiple comparison test.

## Results

### Cortisol and Glucose Levels in Plasma

**Figure 1:**
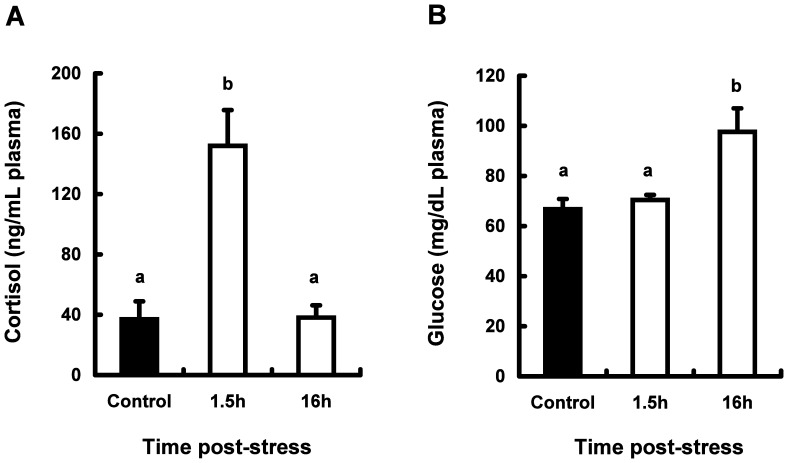
Effect of physiological stress on circulating Cortisol (A) and Glucose (B) levels in plasma from coho salmon *O. kisutch*. Data represent mean ± SEM (*n* = 5). Statistical relationships between groups are indicated by letters where significant differences were detected (*P*<0.05).

The levels of cortisol and glucose in plasma from both stressed and control fish are shown in Fig. 1.

Fish subjected to acute physical stress had plasma levels of cortisol that were significantly elevated, relative to control fish. The plasma cortisol levels were about 4 times higher at 1.5 h after the onset of the stressor than those in control, but had returned to base levels at 16 h post stress (Fig. 1A).

As shown in Fig. 1B, glucose levels in plasma were also significantly elevated at 16 h post stress compared to control fish. Those data about the changes in the levels of both cortisol and glucose indicated that fish were effectively administered stress by the handling.

### GH and IGF-1 Levels in Plasma

**Figure 2:**
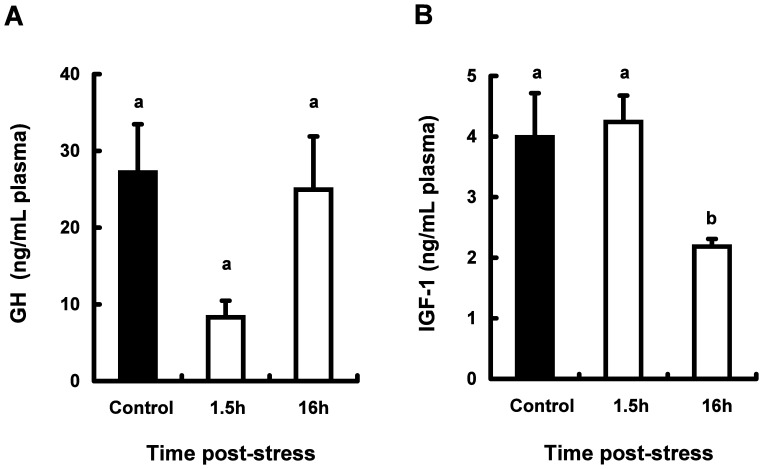
Effect of physiological stress on circulating GH (A) and IGF-1 (B) levels in plasma from coho salmon *O. kisutch*. Data represent mean ± SEM (n = 5). Statistical relationships between groups are indicated by letters where significant differences were detected (p<0.05).

The concentrations of GH and IGF-1 in the plasma from stressed and control fish are shown in Fig. 2.

Plasma GH levels were not significantly different between control and stressed fish (Fig. 2A). As shown in Fig. 2B, plasma IGF-1 concentration of stressed fish at 1.5 h post stress was as same level as control fish. However, at 16 h post stress, plasma levels of IGF-1 in stressed fish decreased significantly compared with the control fish.

### *gh*, *ghr*, and *igf1* mRNA Levels

**Figure 3:**
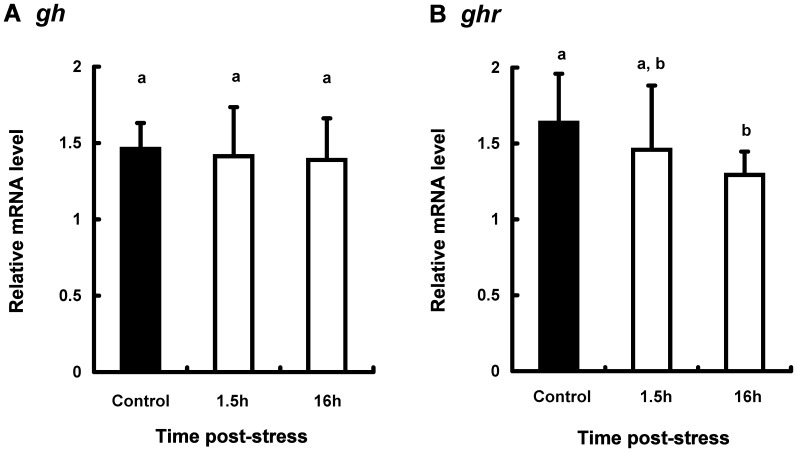
Expression levels of *gh* (A) and *ghr* (B) mRNA in the pituitary from coho salmon *O. kisutch*. The expressions of target gene were normalized by *β-actin* expressions. Data represent mean ± SEM (n = 7). Statistical relationships between groups are indicated by letters where significant differences were detected (p<0.05 for *gh* and p<0.1 for *ghr*).

**Figure 4:**
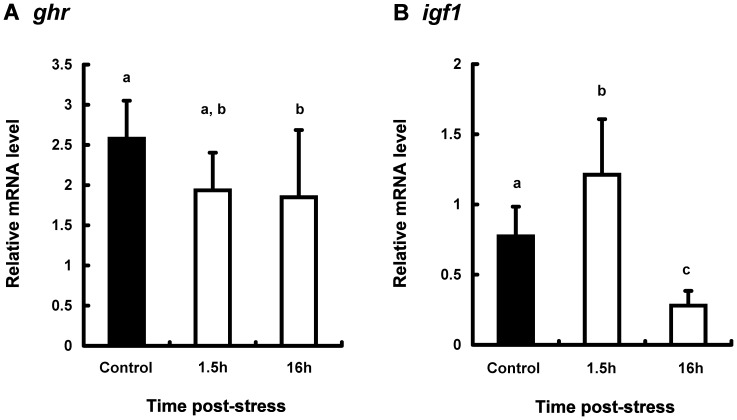
Expression levels of *ghr* (A) and *igf1* (B) mRNA in the liver from coho salmon *O. kisutch*. The expressions of target gene were normalized by *β-actin* expressions. Data represent mean ± SEM (n = 7). Statistical relationships between groups are indicated by letters where significant differences were detected (p<0.05 for *ghr* and p<0.01 for *igf1*).

**Figure 5:**
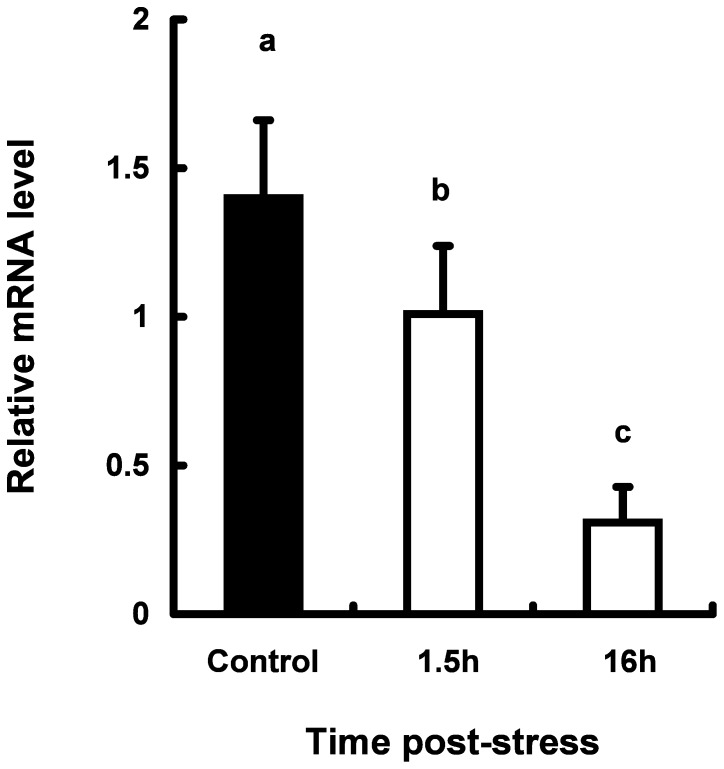
Expression levels of *ghr* mRNA in the muscle from coho salmon *O. kisutch*. The expressions of target gene were normalized by *β-actin* expressions. Data represent mean ± SEM (n = 7). Statistical relationships between groups are indicated by letters where significant differences were detected (p<0.01).

The expression levels of *gh, ghr,* and *igf1* mRNA in the several tissues of stressed and control fish were compared (Fig. 3–5).

In the pituitary, *gh* mRNA levels were not significantly different between control and stressed fish (Fig. 3A). *ghr* mRNA expressions in the pituitary of stressed fish decreased gradually after stress treatment (Fig. 3B).

As shown in Fig. 4A, *ghr* mRNA levels in the liver from stressed fish decreased in a similar manner as that in the pituitary. On the other hand, the hepatic *igf1* mRNA level of stressed fish was significantly higher 1.5 h post-stress relative to the control fish (Fig. 4B). At 16 h post stress, mRNA levels of *igf1* in the liver of stressed fish were observed to decrease significantly compared with the control fish.

*ghr* mRNA expressions decreased gradually after stress treatment in the muscle of stressed fish (Fig. 5). *ghr* mRNA levels declined to 30% of level of control fish 16 h post-stress. On the other hand, *gh* mRNAs in both liver and muscle and *igf1* mRNA in muscle were not detected under the present experimental condition.

## Discussion

The results of this study demonstrated for the first time that acute physiological stress (induced by acute handling stress) can down-regulate the expressions of certain genes related to growth in coho salmon, and also that, a relationship may exist between the neuroendocrine stress response and growth-related gene expression.

The changing pattern of both cortisol and glucose plasma levels in acute-stressed fish observed in the present study are similar to those reported for fish including salmonids [13], [14], [36]. Cortisol levels in unstressed fish are reported to have a wide range [11], [13], [37]. An ideal cortisol level in plasma has been thought to be <10 ng/ml, so that prestress plasma cortisol levels observed in the present study seem to be higher than an ideal cortisol level. However, many environmental and developmental factors (such as maturity, temperature, and time of day) affect resting level of cortisol in fish [11], [13], [37]. In particular, as observed in the present study, juveniles often show high plasma cortisol values even under unstressed conditions [27], [32], [38]–[40].

Pituitary-secreted GH plays an important role in the regulation of many important physiological phenomena in fish, such as growth, osmotic balance, and immune responses. The actions of GH are initiated by its binding to GHR on the cell membrane of target tissues. Accordingly, the pituitary is recognized as a main organ of the GH-IGF-1 axis in fish [41], [42]. The exposure to chronic stress has been observed to cause decreased growth rates in a wide range of fish [11], [12], [15], [16], [25], [26]. This phenomenon might include interactions between glucocorticoids such as cortisol and factors of the GH-IGF-1 axis. In the present study, whereas pituitary *gh* gene expression was not affected, the expression of the *ghr* gene in the pituitary was significantly decreased in response to handling stress. Peterson and Small reported that high levels of exogenous cortisol reduced *gh* mRNA levels pituitary in channel catfish [43]. However the handling-related stress response in the present study was short and plasma cortisol levels returned to normal levels after 16 h. The small change in pituitary *gh* gene expression levels in the present study should be too small to detect as a result. In fact, in the present study, the plasma GH levels observed in stressed fish were not different from those in controls. This phenomenon might also be concerned with the sensitivity of GH to cortisol. Although *ghr* gene expression and the binding activity of GH to GHR have been detected in several tissues in fish, most studies on fish GHR have been conducted with liver, gill, and muscle. However, understanding of the regulatory properties of *ghr* gene expression in the fish pituitary is lacking. Although GH binding with GHR in the brain has been observed to be low, a negative feedback function might be one of the roles of GH and GHR binding in the brain [1], [3]. How physiological stress, as imposed in this study, affects the regulation of *ghr* gene expression in the pituitary of fish remains unknown in fish and further study is needed.

Hepatic *igf1* expression rapidly increased at 1.5 h post stress without a change in *ghr*, whereas both *ghr* and *igf1* levels decreased at 16 h after the treatment of handling stress in this study, indicating that the expression of *gh*, *ghr*, and *igf1* genes responded differently to physiological stress. Accordingly, the expression of *igf1* gene seems to have been independently affected by stress other than GH and its signal transduction through GHR. Actually, seasonal changes between GH and IGF-1 in fish plasma can differ by several weeks or more [7], [44]. In the present study, although the levels of plasma GH were unchanged by the stressor, plasma IGF-1 concentration was decreased by handling stress. In tilapia, no change in plasma GH levels has been observed after confinement stress, whereas a transient increase in plasma IGF-1 levels occurred at 0.5 h post-stress. This transient changes in plasma IGF-1 in response to stress are thought to concern with hemodynamic effects on release of IGF-1 from the liver [28], [45]. Those observations suggest that plasma IGF-1 levels may not be directly regulated by the circulating GH levels alone. In practice, GH injection has also been observed not to affect the *igf1* expression in the gill of the sea bream [46]. The difference in the actions of fish GH and IGF-1 suggest that tissue-specific regulation of *igf1* expression by GH and other physiological conditions might exist in fish [2], [3], [7], [28], [46].

Handling stress administered in this study caused *ghr* mRNA levels in both liver and muscle to decrease. As a result, the amount of GHRs in both liver and muscle might be reduced. The number of GHR in fish tissue has been reported to be affected by glucocorticoids, somatostatin, fasting, and heat stress [5], [14], [47]. In mammalian hepatocytes, high levels of glucocorticoids have been shown to inhibit GH-induced *igf1* gene expression and also reduce *ghr* mRNA level [48]. Hepatic *ghr* and *igf1* gene expression was reduced in stressed fish, whereas both circulating GH level and pituitary *gh* mRNA level were not affected in this study. These phenomena suggest that, assuming similar levels of GH secretion, circulating GH level was maintained even after the acute stress, but the binding capacity of GH to GHR and sensitivity to GH may have been reduced. The reduction of GHR level in the liver and muscle of stressed fish would also decrease the action of GH to the liver and muscle. Accordingly, the elevation of circulation glucocorticoids such as cortisol may have induced the down-regulation of both *ghr* and *igf1* gene expressions and resulted in the decrease of plasma IGF-1 levels in stressed coho salmon. The decrease of IGF-1 level in plasma at 2–24 h post stress has often been observed in many fish species [28]. Our observations about the changes in circulating IGF-1 and hepatic *igf1* gene expression levels in stressed fish are in agreement with those reported for fish administered exogenous cortisol and GH [27], [49]–[52]. The hormonal regulation of hepatic *igf1* gene expression can vary depending on the kinds of hormones, clearance rates of hormones in plasma, and species, especially, regarding regulation of *igf1* expression in liver by glucocorticoid. Unfortunately, mRNA expressions of *igfI* in the muscle and *gh* in both liver and muscle were not detected under the present experimental conditions. The expression level of *gh* in extrapituitary tissues of fish is known to be very low [53]. On the other hand, IGF-1 and *igfI* are known to be expressed and to act at both the endocrine (hepatic) and local (paracrine and autocrine) levels. Indeed, *igfI* has been detected in several tissues, such as brain and muscle, although liver is the primary site of IGF-1 production in fish [2], [7], [28], [53], [54]. Accordingly, it is necessary to examine the experimental condition to detect *igfI* in the tissues other than liver, and further study is needed to know the change in locally produced IGF-1 and expressed *igfI* in the tissues of acute-stressed fish.

Consistent with our findings, chronic stress and exogenous cortisol have been reported to reduce the growth of fish, and also influences metabolism through the action of glucocorticoid via the hypothalamus-pituitary-interrenal (HPI)-axis [9], [47]. Direct evidence for the effect of physiological stress on the relationship between IGF-1 and growth in fish has been limited [7], [28]. In addition to the results of the present study, we recently observed that growth-related gene expressions in coho salmon were affected by heat stress, but returned to control levels at 48 h post-stress [55]. The pattern of gene expression in response to an acute stress in trout liver was determined by cDNA microarray [13], [56]. In the study, while transient changes in genes were observed within 3 h post-stress, a few genes remained elevated at around 24 h post stress. Hence, the change in the expression of GH-IGF-1 axis-related genes and growth in response to stressors over prolonged periods should be studied. Nutritional status has been reported to affect the expression of growth-related factors in fish [2], [6]–[8], [27]–[30], [57]. We have observed that an antioxidative supplement can dramatically reduce the stress-induced damage in fish [23], [24], [58], [59]. Accordingly, there is need for further study of the beneficial effects of nutraceutical supplements on growth-related factors in stressed fish.

In conclusion, the present work is expected to provide significant information concerning both an applied and basic aspects, such as an improvement of the fitness in cultured fish. Additionally when we treat fish as an experimental animal, the present results also suggest that we should keep in mind the possible effects of acute stress derived from handling on the expressions of growth-related factors in fish. Further investigation is required to elucidate fully the relationships among physiological stress, growth-related factors, and growth in fish.
